# Transfer of Aging: Implications for Pediatric Solid Organ Transplantation

**DOI:** 10.1111/petr.70226

**Published:** 2025-11-26

**Authors:** Rosalie Wolff von Gudenberg, Lucas Said Josef Eckholt, Simon Moosburner, Dustin Greve, Leonard Boerger, Kilian Walter, Leonhard Wert, Dominik Geiger, Adam Penkalla, Jan D. Schmitto, Maximilian Y. Emmert, Arjang Ruhparwar, Nian Yeqi, Stefan G. Tullius, Jasper Iske

**Affiliations:** ^1^ Division of Transplant Surgery, Department of Surgery Brigham and Women's Hospital, Harvard Medical School Boston Massachusetts USA; ^2^ Department of Cardiothoracic, Transplantation and Vascular Surgery Hannover Medical School Hannover Germany; ^3^ Department of Cardiothoracic and Vascular Surgery Deutsches Herzzentrum der Charité (DHZC), Augustenburger Platz 1 Berlin Germany; ^4^ Charité‐Universitätsmedizin Berlin Corporate Member of Freie Universität Berlin and Humboldt‐Universität Zu Berlin Berlin Germany; ^5^ Department of Surgery Campus Charité Mitte, Campus Virchow‐Klinikum, Experimental Surgery Charité‐ Universitätsmedizin Berlin, Corporate Member of Freie Universität Berlin and Humboldt Universität Zu Berlin Berlin Germany; ^6^ Institute for Regenerative Medicine (IREM) University of Zurich Zurich Switzerland; ^7^ Tianjin Key Laboratory for Organ Transplantation Nankai University Tianjin China; ^8^ Berlin Institute of Health at Charité‐ Universitätsmedizin Berlin BIH Biomedical Innovation Academy, BIH Charité Clinician Scientist Program Berlin Germany

## Abstract

Solid organ transplantation (SOT) is a life‐saving intervention for pediatric patients with end‐stage organ failure. Due to the limited availability of pediatric donor organs, organs from older donors are frequently utilized, increasing the risk of age‐mismatched transplants. Older donor organs are linked to heightened immunogenicity, rejection rates, and impaired long‐term outcomes. Emerging evidence suggests that aged donor organs may transfer senescence to pediatric recipients, accelerating aging‐like processes such as frailty, cognitive decline, and organ dysfunction. Additionally, the induction of senescence could alter pediatric conditions like chronic kidney disease (CKD), juvenile idiopathic arthritis (JIA), and pediatric brain tumors which have been linked to augmented senescence. Animal models have shown that older donor organs induce senescence‐associated changes in young recipients, including immune dysfunction and physical and cognitive impairments. This review highlights the role of cellular senescence in pediatric organ transplantation and discusses strategies to mitigate its impact. Therapies targeting senescence, such as senolytics, offer a potential approach to improve outcomes in pediatric recipients. Further research is needed to validate these findings in human studies and guide clinical strategies that expand the donor pool while prioritizing age‐matched transplantation for pediatric patients.

## 1 Introduction

Solid organ transplantation (SOT) for pediatric patients is the preferred treatment for most children with end‐stage organ failure and represents the primary life‐saving option. However, the scarcity of pediatric donor organs presents a significant challenge in meeting this demand. Due to this shortage, pediatric patients frequently receive organs from older, adult donors. Although donor age constitutes a significant risk factor for transplant outcomes with older organs displaying the highest rejection rates [1, 2], the utilization of such organs remains of critical relevance considering the insufficient supply of pediatric donor organs [3]. Consequently, heterochronic transplantations with donor/recipient age‐discrepant combinations have become a clinical routine in pediatric patients.

It has been shown that senescent cells accumulate with aging and have been identified as critical for promoting the immunogenicity of older organs, associated with the accumulation of cell‐free mitochondrial DNA that accelerates alloimmune responses [4].

Cellular senescence is characterized as a stable and terminal state of growth arrest based on acquired anti‐apoptotic pathways, which render senescent cells resistant to apoptosis. Thus, senescent cells accumulate in many tissues with aging [5]. Senescent cells exhibit a pronounced pro‐inflammatory secretome consisting of cytokines (IL‐6, IL‐8, TNF‐α), chemokines (CCL2, CCL20), and matrix remodeling enzymes, referred to as the “Senescent Associated Secretory Phenotype” (SASP). The production of SASP is a cardinal feature of senescent cells, leading to sterile inflammation in tissues, which in turn contributes to age‐related tissue dysfunction, chronic age‐related diseases, and organismal aging, impairing tissue homeostasis and hindering neighboring cell function [6].

In this review, we aim to explore the potential role of cellular senescence in age‐disparate organ transplantation in pediatric patients, focusing on its implications for long‐term outcomes and the unique challenges associated with this vulnerable population. While direct transplantation studies are limited [7, 8], the concept is further strongly supported by mechanistic insights from heterochronic parabiosis, blood exchange, and SASP‐mediated senescence induction [9, 10, 11, 12, 13]. Multiple studies show the induction of senescence in young recipients through SASP factor mediation after the transplantation of senescent cells [14] for instance, into the skin of young recipients [15] or into knee joints [16].

Beyond transplantation, cellular senescence has also been implicated in various pediatric diseases, including chronic inflammatory conditions [17, 18, 19, 20, 21].

## 2 Role of Cellular Senescence in Pediatric Diseases

Senescence is traditionally viewed as a cellular response to stress or replicative exhaustion, acting as a safeguard to prevent the proliferation of damaged cells. As such, it is widely recognized as a hallmark of aging and a driver of age‐related diseases [22, 23, 24, 25, 26, 27, 28]. However, accumulating evidence suggests that senescence is not limited to aging tissues but also plays a role in pathological conditions across all stages of life.

Interestingly, senescence is not only a hallmark of aging but also a conserved developmental process. During embryogenesis, transient senescent cells appear at specific sites to facilitate tissue remodeling, demonstrating that senescence is an evolutionarily adapted mechanism [22]. While this process is tightly regulated during development, its persistence or dysregulation in early life may contribute to pediatric diseases, as chronic senescence with accumulating senescent cells drives sustained inflammation through the SASP [29] (Table 1).

**TABLE 1:** Key studies delineating the role of senescence in pediatric diseases.

Major finding	Authors (year)	Study design	Relevance
Senescence in TEC drives tubular dysfunction and early CKD in pediatric populations	Knoppert et al. [17]	Retrospective cohort study with translational analysis	First study linking biopsy‐proven senescence with CKD progression in this patient group
Macrophage senescence in glioma contributes to tumor initiation and progression	Li et al. [18]	Review/mechanistic exploration	Focuses on brain macrophage reprogramming in glioma
Senescence in pituitary stem cells drives pediatric craniopharyngioma	Gonzalez‐Meljem et al. [30]	Mouse model + human data	Demonstrates SASP‐driven paracrine tumorigenesis
Premature T cell senescence observed in pediatric onset MS (POMS)	Balint et al. [21]	Immunophenotyping study	Changes in T‐cell compartment in POMS patients, resembling those of 20–30 years older controls
T‐cell senescence in Juvenile Idiopathic Arthritis (JIA)	Dvergsten et al. [31]	Flow cytometry + functional assays	CD8+ T cells exhibit senescence and pro‐inflammatory cytokine profile
Cellular senescence present in biliary atresia and pediatric liver fibrosis	Jannone et al. [32]	Histopathology + transcriptomics	Highlights senescence as fibrosis driver in pediatric hepatology
Senescence detected in livers of pediatric ESLD patients	Gutierrez‐Reyes et al. [33]	Histological study	Confirms senescence markers in pediatric endstage liver disease

Recent studies have identified senescent cells in various pediatric disease contexts, implicating them in developmental disorders, chronic inflammatory conditions, and an increased predisposition to long‐term organ dysfunction [17, 18, 20, 34]. While cellular senescence can serve beneficial functions—such as limiting fibrosis and promoting tissue repair—its dysregulation in early life may contribute to disease progression rather than protection.

### 2.1 Chronic Kidney Disease

Cellular senescence in tubular epithelial cells (TECs) has emerged as a driver of tubular dysfunction and early chronic kidney disease (CKD) in pediatric populations, particularly among childhood cancer survivors exposed to nephrotoxic therapies [17]. This pathological process is characterized by the accumulation of senescent TECs exhibiting hallmarks such as cell‐cycle arrest linked to the upregulation of p21, DNA damage, and mitochondrial dysfunction, which collectively impair renal repair mechanisms and promote fibrosis [31, 35]. In pediatric oncology patients, kidney biopsies reveal TECs with enlarged nuclei and robust p21 expression, directly linking senescence to functional declines such as polyuria, low‐molecular‐weight proteinuria, and reduced glomerular filtration rate (GFR) [17, 35]. These findings are further supported by genetic studies implicating defective DNA damage repair pathways—such as FAN1 mutations, which disrupt interstrand cross‐link repair and exacerbate senescence‐associated renal fibrosis—in CKD progression [36]. Chemotherapeutic agents like ifosfamide amplify this risk by inducing mitochondrial damage and oxidative stress, leading to irreversible tubular injury in up to 50% of treated patients, with 10 out of 34 progressing to end‐stage renal disease in recent clinical cohorts [37, 38].

Emerging therapeutic strategies targeting senescence, including senolytics, Klotho supplementation, and mTOR inhibitors, show promise in preclinical models for mitigating fibrosis and preserving renal function [31, 39]. However, the long‐term management of pediatric CKD requires early detection of senescence markers (e.g., SA‐β‐gal, p16) and tailored interventions to address the unique vulnerability of developing kidneys to age‐related molecular insults.

### 2.2 Senescence in Pediatric Brain Tumors and Neurological Diseases

Beyond renal pathology, senescence has been implicated in pediatric neuro‐oncology, particularly in the development of pediatric low‐grade gliomas (pLGG), the most common central nervous system (CNS) tumors in children [18]. In pediatric low‐grade gliomas (pLGG), oncogene‐induced senescence (OIS) is a key feature of tumor biology. Recent multi‐omics analyses of BRAF‐driven pLGG models demonstrated that OIS and the associated SASP program are directly regulated by MAPK signaling, with senescent tumor cells expressing high levels of IL‐1B, IL‐6, IL‐8, MMPs, and other SASP factors [40]. While OIS imposes a proliferative barrier on tumor cells, SASP secretion simultaneously remodels the tumor microenvironment, potentially supporting disease maintenance and progression [40]. This dichotomy illustrates the distinction between *acute/transient senescence*, which is protective and self‐limiting (e.g., OIS acting as a growth arrest mechanism), and *chronic/persistent senescence*, which sustains SASP‐driven inflammation and fibrosis and may foster tumor progression.

In addition to gliomas, cellular senescence has also been implicated in the tumorigenesis of other pediatric brain tumors, such as Pituitary adenomas (PAs) highlighting its broader relevance in pediatric neuro‐oncology [19].

In human tissue of adamantinomatous craniopharyngioma (ACP), a pediatric pituitary tumor, epithelial cells—particularly those forming whorl‐like structures—were shown to undergo β‐catenin‐driven senescence [41]. These senescent cells express hallmarks such as p21, activation of DNA damage response pathways, and G1/S cell cycle arrest, and secrete a senescence‐associated secretory phenotype (SASP). SASP exerts paracrine effects on neighboring epithelial, glial, and myeloid cells, promoting proliferation of non‐senescent epithelial cells, remodeling of the extracellular matrix through MMPs and fibronectin/laminin modulation, as well as angiogenesis and tissue invasion via factors such as VEGF and SPP1. Spatial transcriptomics and single‐cell analyses reveal that SASP‐secreting senescent cells are present not only within epithelial whorls but also in palisading epithelia, glial compartments, and macrophage populations, establishing them as central modulators of the tumor microenvironment [41].

Similarly, murine models have demonstrated that β‐catenin‐driven senescence in pituitary stem cells initiates paracrine tumorigenesis via SASP‐mediated activation of MEK/ERK pathways in adjacent cells [42]. These senescent clusters exhibit γH2A.X foci, NF‐κB activation, and IL‐6/IL‐1α secretion, fostering extracellular matrix remodeling and invasive growth [42]. Together, these studies identify senescence‐induced SASP signaling as a key driver in shaping the ACP tumor microenvironment and facilitating disease progression [41, 42].

Collectively, senescence has been linked to tumorigenesis and tumor growth in pediatric brain tumors. However, these insights are largely derived from preclinical models and correlative analyses in patient samples. A direct causal role of senescence and SASP in initiating or sustaining pediatric brain tumor growth has not yet been firmly established, and it remains uncertain to what extent these mechanisms apply across the diverse molecular subtypes of pLGG and ACP. Further studies, particularly in larger patient cohorts and advanced preclinical models, are therefore required to substantiate the clinical relevance of the SASP‐driven tumorigenesis in pediatric neuro‐oncology.

### 2.3 Premature Immune Senescence in Pediatric Multiple Sclerosis and Juvenile Idiopathic Arthritis

Senescence also plays a role in pediatric immune‐mediated diseases, as seen in pediatric‐onset multiple sclerosis (POMS). POMS represents a unique model of accelerated immune aging, with the disease itself driving premature senescence in the T‐cell compartment [20, 21]. While multiple sclerosis (MS) has traditionally been viewed as an imbalance between effector and regulatory immune responses, emerging evidence suggests that POMS patients exhibit profound age‐inappropriate immunosenescence [20]. Senescence in neural progenitor cells may limit remyelination, a critical process for maintaining neural function in MS patients [30, 43]. Additionally, children with POMS have been found to have shorter telomeres, suggesting that MS is associated with biological aging. The ratios between naive and memory T cells in POMS patients mirror those seen in adult‐onset MS, resembling those of individuals 20 to 30 years older, which may be attributed to early thymic involution [21]. Premature immune senescence thus appears to contribute to POMS pathology and disease progression. However, its causal role in driving disease onset and long‐term progression remains to be firmly established.

A similar pattern of immune senescence has been observed in juvenile idiopathic arthritis (JIA), the most common chronic rheumatic disease in children. Accelerated immune aging in JIA has been linked to T‐cell senescence and disruptions in T‐cell homeostasis [44]. Senescent T cells in JIA, characterized as CD31 + CD28(null) CD8+ T cells, exhibit limited mitotic capacity, express high levels of senescence markers such as histone γH2AX and p16, and display shortened telomeres [34]. These cells are highly enriched in the synovial fluid of affected joints, where they can constitute up to 80% of all αβ T cells, compared to much lower frequencies in healthy children. Their presence is closely linked to inflammatory processes, as they produce high levels of pro‐inflammatory cytokines such as IL‐17A, IL‐6, IFNγ, and TNFα, thereby actively driving and sustaining joint inflammation in JIA [45]. Of relevance, reduced thymic output in JIA patients is thought to drive compensatory autoproliferation of T cells, ultimately leading to premature senescence [44].

These findings position senescence as a unifying mechanism across diverse pediatric diseases, influencing organ dysfunction, tumorigenesis, and immune dysregulation. As research advances, targeting senescence may offer novel therapeutic opportunities to improve long‐term health outcomes in pediatric patients. Beyond its role in pediatric disease, recent studies suggest that the transplantation of older organs may induce a senescent phenotype in young recipients, which may in turn exacerbate disease progression [7, 8, 46, 47, 48, 49].

## 3 The Impact of Age‐Mismatched Organ Transplantation in Pediatric Patients

**FIGURE 1:**
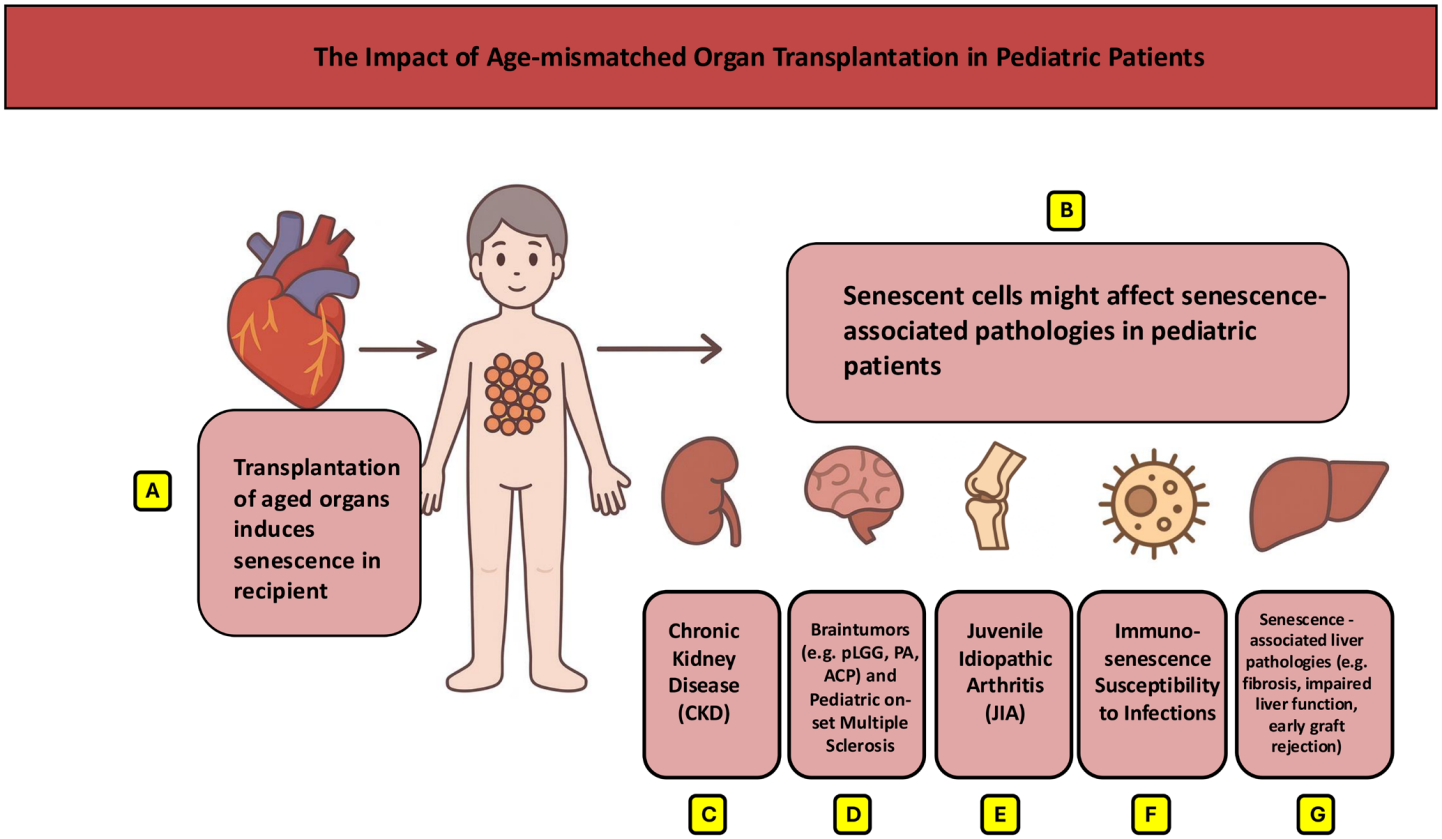
The Impact of age‐mismatched organ transplantation in pediatric patients. Increasing evidence suggests that (A) transplanting older organs into younger recipients could induce senescence in recipient tissues with augmented frequencies of senescent cells. Since the age‐mismatch is most pronounced in pediatric transplantation this phenomenon could be even more relevant for those patients. (B) Various pediatric pathologies in turn have been linked to senescence as an underlying pathomechanism which may in turn be accelerated through senescence induction following age‐mismatched transplantation.

The impact of age‐mismatched organ transplantation in pediatric patients is a critical area of concern, as donor age significantly influences transplant outcomes. Clinical evidence consistently demonstrates that utilizing older donor organs is associated with inferior graft survival compared to using organs from younger donors. For instance, studies on pediatric split‐liver transplants have shown significantly better graft survival rates in recipients who received organs from younger donors (under 45 years old) compared to those receiving organs from older donors (over 45 years old) [50]. Similarly, age‐disparate pediatric heart transplantation settings have noted decreased survival rates, particularly in recipients between 11 and 17 years old [51], and an increased risk of graft loss has been demonstrated in pediatric kidney graft recipients receiving kidneys from older donors [46]. While these clinical observations highlight the negative impact of donor age, emerging mechanistic studies suggest that this effect is at least partially mediated by the induction of cellular senescence in young recipients [4, 7, 8, 49] (Figure 1).

In the context of renal transplantation, young mice receiving aged kidneys exhibited increased accumulation of p16‐positive senescent cells, which significantly exacerbated interstitial fibrosis and tubular atrophy, resulting in nephron loss, impaired renal function, and reduced long‐term graft survival [8, 52] findings that provide direct experimental support for the concept of “transfer of aging” from donor to recipient. This is particularly relevant in pediatric settings, as senescent TECs are well‐established drivers of chronic kidney disease (CKD) [25, 35, 53]. Senescent TECs secrete TGF‐β, IL‐6, and CCL2, which promote epithelial‐to‐mesenchymal transition (EMT) and activate fibroblasts, leading to collagen deposition and interstitial fibrosis. This may be exacerbated in heterochronic transplants, where older donor kidneys introduce pre‐senescent TECs that secrete SASP factors, which promote fibrosis and tubular atrophy, processes known to contribute to nephron loss and potentially accelerate progression to end‐stage renal disease (ESRD) [54, 55, 56, 57]. Given the systemic effects of SASP, it is plausible that the transplantation of other aged organs—such as the heart or liver—could similarly contribute to CKD onset and progression in pediatric recipients.

In young recipients of aged donor livers, premature senescence in liver tissue has been linked to an increased risk for hepatobiliary disease development [7, 58]. Transplantation of aged organs into young recipients in rodent models has been shown to induce the accumulation of senescent cells in the liver, as evidenced by increased expression of senescence markers such as p16 in recipient liver tissue, which has also been linked to higher rates of fibrosis, impaired liver function, and early graft rejection [7, 59, 60].

Premature senescence is already recognized as a key driver of deleterious liver remodeling and dysfunction in adult hepatobiliary diseases, and recent studies have further shown that it also plays a central role in pediatric conditions such as biliary atresia (BA) [58] and end‐stage liver disease (ESLD). For example, pediatric patients with end‐stage liver disease (ESLD) exhibit senescence markers such as SA‐β‐gal, p53, p21, and p16 in the Canals of Hering, interlobular bile ducts, and hepatocytes surrounding regenerative nodules in cirrhotic livers [32].

Consequently, the transplantation of aged organs, including the liver itself, into pediatric recipients may promote the accumulation of senescent cells in the liver, thereby increasing the risk for the development of senescence‐associated liver diseases [7, 47, 58].

The systemic effects of the SASP can induce senescence in distant tissues. SASP factors, secreted by senescent cells, have both local and systemic impacts, spreading senescence to previously healthy cells throughout the body and promoting chronic inflammation [33, 61, 62]. This pro‐inflammatory milieu has been shown to impair beta cell function and survival in pancreatic islets, thereby facilitating the development of metabolic disorders such as type 1 diabetes [7, 63]. Experimental models further demonstrate that the clearance of senescent beta cells is sufficient to prevent autoimmune‐mediated beta cell destruction and halt disease progression [63].

In a similar vein, the systemic induction of senescence in young mice receiving aged tissue can significantly contribute to early‐onset frailty and impaired tissue regeneration. For example, elevated expression of the senescence marker p16 in femoral muscle has been shown to correlate with diminished physical performance in young animals receiving aged tissue, closely resembling age‐related functional decline [7]. Furthermore, transplantation of senescent preadipocytes into the abdominal cavity of young mice not only promoted frailty but also induced senescence in neighboring healthy cells [14], illustrating the paracrine spread of senescence. Heterochronic blood exchange experiments further support these findings, demonstrating that even a single exposure to old blood can induce senescence in multiple tissues of young animals, driven by systemic factors such as the SASP [49]. This widespread senescence has particularly detrimental effects on stem cell‐dependent tissues: in murine models, skeletal muscle regeneration was impaired due to reduced self‐renewal of muscle satellite cells [64], while in progeroid mice with BubR1 deficiency, senescence in muscle and fat progenitor cells led to pronounced sarcopenia and adipose tissue loss [65], further illustrating the profound impact of systemic senescence on tissue homeostasis and organismal vitality [66, 67].

Following transplantation of aged organs into young recipients, not only organ‐specific effects but also systemic impacts on immune cell compartments have been observed. In particular, old‐to‐young transplantation has been linked to features of immunosenescence, including T cell dysfunction [7, 34, 48, 68] and increased secretion of pro‐inflammatory SASP factors [7].

One study in old kidney transplant recipients demonstrated accelerated T cell senescence within the first year post‐transplant, characterized by shortened T cell telomere length and accumulation of these senescent CD57^+^CD28^−^ T cells [48, 68]. Similarly, kidney transplant recipients with long‐term graft survival (> 10 years under CNI monotherapy) exhibited increased frequencies of senescent CD27^−^CD28^−^CD45RO^+^CD8^+^ T cells with innate‐like functional features and augmented perforin expression, mediating T cell‐derived inflammaging [47]. Another study highlighted, how young recipients of aged organs exhibited elevated levels of SASP factors, including eotaxin 1 [7], a pivotal chemokine crucial for eosinophil homing to the lungs of asthmatic patients [69]. Notably, severe therapy‐resistant asthma (STRA), a condition characterized by chronic inflammation and premature cellular aging, is associated with similar senescence‐related features, including shortened telomeres and elevated plasma eotaxin‐1 [70, 71]. In a cohort of 267 children, STRA patients showed significantly shorter telomeres compared to mild asthma and healthy controls, with nearly twofold higher plasma eotaxin‐1 levels and an inverse correlation between telomere length and eotaxin‐1.

Similarly, young recipients of aged kidneys develop a senescent T cell phenotype, characterized by loss of CD28 and shortened telomeres in CD8^+^ T cells, closely mirroring the features observed in juvenile idiopathic arthritis (JIA), where oligoarticular patients (*n* = 62) show elevated frequencies of CD31^+^CD28^−^ CD8^+^ T cells with reduced proliferative capacity and expression of senescence markers γH2AX and p16 [34].

Together, these findings suggest that senescence induced by aged organs could theoretically contribute not only to organ‐specific dysfunction but also to systemic immune aging, potentially increasing the risk for inflammatory and asthma‐like phenotypes in pediatric recipients. While direct clinical evidence linking donor organ age to asthma and JIA incidences in pediatric patients post‐transplantation is currently lacking, these parallels highlight a hypothesis that warrants further investigation.

Systemic senescence can also affect the brain, altering neurological functions and potentially contributing to neuropathologies in pediatric patients. In a murine model, it was shown that heterochronic heart transplantation (HTX) can induce senescence in the recipient brain through SASP signaling [7]. In this model, young recipients of aged hearts exhibited increased systemic levels of SASP factors, including circulating mitochondrial DNA (mtDNA) [4]. Injection of isolated mtDNA in young mice recapitulated these effects, inducing similar elevations in inflammatory SASP factors alongside upregulation of senescence markers p16 and p21 in the hippocampus area of the brain, which was further linked to functional deficits, such as a decline in cognitive capacity, augmented anxiety, and compromised spatial working memory. Together, these findings suggest that age‐mismatched organ transplantation may contribute to brain senescence and inflammaging via SASP [7].

Supporting this, intradermal transplantation of senescent skin fibroblasts into young mice similarly showed brain senescence induction [15]. After transplantation, recipients showed in the hippocampal CA3 region an increase of p21‐positive cells, elevated SASP signals (Il‐1α, Il‐6), alongside cognitive deficits in Y‐maze and Stone's T‐maze. Plasma profiling also revealed systemic IL‐6 elevation, and tissue analyses showed tissue‐specific p16 and p21‐positive senescence cells. These findings further indicate that transplantation of senescent cells into young recipients leads to senescence induction and aging in the brain.

Notably, SASP‐driven mechanisms have also been implicated in the development of pediatric brain tumors like APC [41, 42] and pediatric low‐grade gliomas (pLGG) [40]. For instance, in a mouse model of adamantinomatous craniopharyngioma (ACP), senescent epithelial cells secrete a senescence‐associated secretory phenotype (SASP) that stimulates the proliferation of neighboring non‐mutant epithelial cells, thereby driving tumor formation through paracrine mechanisms [41, 42]. These SASP‐secreting cells act as signaling hubs, reshaping the tumor microenvironment and promoting disease progression [42].

In a similar manner, the role of SASP has also been described in the development of pLGG. Senescent tumor cells secrete a SASP that generates a pro‐inflammatory and tissue‐remodeling microenvironment, stimulating neighboring cells and thereby supporting tumor progression [40].

Consequently, in the setting of heterochronic transplantation, where senescence is induced in the recipient through the engraftment of an aged organ, the local activation of SASP could create a permissive microenvironment that may support tumor initiation and progression. However, a direct causal link between heterochronic transplantation and pediatric brain tumor development has not been demonstrated to date; rather, this remains a hypothesis based on mechanistic parallels between transplant‐induced senescence and senescence observed in pediatric brain tumors.

Taken together, these findings outline a profound and multifaceted impact of donor organ age on pediatric transplant recipients with the potential to alter the susceptibility to specific diseases and pathology development.

Despite these risks, the use of older donor organs remains clinically relevant, particularly in pediatric transplantation where organ scarcity is a major limiting factor. The number of patients on transplantation waiting lists continues to rise, while the availability of standard criteria donor (SCD) organs for pediatric recipients [72, 73] remains stagnant or even declines [74]. The utilization of expanded criteria donor (ECD) and donation after circulatory death (DCD) organs helps to bridge this gap and increases the chances for patients to receive a life‐saving transplant [74].

For instance, in children with end‐stage kidney disease, a major driver is the persistent shortage of size‐ and age‐matched pediatric donor organs, which leads to prolonged waiting times [75, 76]. Extended dialysis in this population is associated with impaired growth, delayed development, and increased morbidity and mortality. Transplantation with adult donor kidneys, even in very young or low‐weight recipients, has been shown to achieve favorable short‐ and long‐term outcomes when performed with appropriate surgical approaches, such as the extraperitoneal technique, and is not associated with higher rates of delayed graft function, surgical complications, or reduced graft survival compared to larger recipients [75, 77]. Thus, the use of older or expanded criteria donor kidneys offers a viable strategy to bridge the gap between organ supply and demand, ensuring timely access to transplantation and improving overall prognosis in pediatric patients.

Moreover, technological advances such as ex vivo machine perfusion further support the safe utilization of marginal or older donor organs in pediatric transplantation. By minimizing cold ischemic time and enabling prolonged preservation and transport, these approaches effectively expand the donor pool for children with end‐stage organ failure [78]. Recent single‐center experience with the Organ Care System in pediatric heart transplantation demonstrated excellent short‐term graft function and survival, even in complex recipients, underscoring that innovative preservation strategies can mitigate risks traditionally associated with non‐standard donor organs while improving access to life‐saving transplants [79].

## 4 Outlook and Future Perspectives

Current evidence for senescence transfer in age‐mismatched transplantation remains predominantly derived from murine models. While these studies demonstrate that aged organs propagate senescence via SASP factors and impair physical and cognitive function in young recipients, human validation is urgently needed, as animal models may not fully recapitulate the heterogeneity of pediatric transplant populations, including differences in age, underlying disease, immunosuppressive regimens, and developmental stage of the immune system. Such confounding factors highlight the necessity of carefully designed clinical studies to clarify the true relevance of senescence transfer in pediatric transplantation. Longitudinal cohort studies tracking senescence accumulation in pediatric recipients of older versus age‐matched grafts could provide valuable insights into senescence acceleration and its clinical impact. At present, the early detection of senescence markers such as SA‐β‐Gal and p16 requires tissue sampling and is therefore not feasible in daily practice. Future research should focus on the development and validation of non‐invasive biomarkers—such as circulating SASP components or cell‐free mitochondrial DNA—that may enable dynamic monitoring of senescence burden in pediatric transplant recipients [80, 81]. Recent work has already demonstrated the feasibility of such approaches, with SASP‐based biomarker panels from senescent monocytes successfully predicting frailty and other age‐related outcomes in large human cohorts, highlighting their translational potential for future application in pediatric transplant recipients [70].

Given the potential risks of senescence transfer, strategies to mitigate its effects are crucial—particularly in pediatric recipients, who are at heightened risk of premature multimorbidity. Senolytic and senomorphic drugs have emerged as promising candidates to target cellular senescence. Senolytics offer a targeted approach to eliminating senescent cells by inhibiting senescence‐related anti‐apoptotic pathways [82]. Encouragingly, recent studies indicate that senolytic treatments can improve transplant outcomes by promoting senescence clearance [7]. In preclinical models, a single dose of Dasatinib/Quercetin (D + Q) in old donors reduced senescent cell burden improving cardiac allograft survival to levels comparable to young donor organs [4]. Moreover, it was shown that senolytics cleared residual senescent cells in young recipients, restoring muscle satellite cell function and reducing fibrosis in kidney and liver grafts [10, 59]. Additionally, senolytics have been shown to prevent systemic senescence induction in young mice receiving old blood [49] and mitigate mitochondrial DNA release following ischemia–reperfusion injury [4, 83]. Despite these promising observations, the translation of senescence‐targeting therapies into pediatric transplantation requires careful consideration. Senescent cells can also play beneficial roles in tissue repair and development [61, 84], and their indiscriminate depletion has been associated with adverse effects in preclinical models [85]. Furthermore, robust clinical data, particularly in pediatric populations, are lacking. Therefore, while senolytics and senomorphics represent an exciting therapeutic avenue, their application in children must be approached cautiously and supported by rigorous clinical trials. Clinical trials investigating risk profiles and dose efficiency of senolytics drugs are needed.

## 5 Conclusion

Heterochronic transplantation offers a life‐saving therapeutic option for terminally diseased pediatric patients. Increasing evidence suggests that this procedure is associated with the induction of senescence in recipients which in turn may aggravate transplant outcomes. Moreover, the induction of senescence may promote further pediatric pathologies involving fibrotic remodeling, immunosenescence or tumorigenesis which could pose a risk for long‐term outcomes of pediatric transplant patients if confirmed clinically.

Future research should prioritize longitudinal studies that track senescence biomarkers to delineate the clinical impact of senescence induction in pediatric transplant recipients. By recognizing senescence as a modifiable risk factor, clinicians can adopt targeted strategies such as senolytics or senomorphic drugs to mitigate its impact, ultimately improving long‐term outcomes.

## Conflicts of Interest

The authors declare no conflicts of interest.

## Data Availability

Data sharing not applicable to this article as no datasets were generated or analyzed during the current study.
